# DNA repair kinetics in SCID mice Sertoli cells and DNA-PKcs-deficient mouse embryonic fibroblasts

**DOI:** 10.1007/s00412-016-0590-9

**Published:** 2016-05-02

**Authors:** Emad A. Ahmed, Eukene Vélaz, Michael Rosemann, Klaus-P. Gilbertz, Harry Scherthan

**Affiliations:** 1 Laboratory of Immunology and Molecular Physiology, Zoology Department, Faculty of Science, Assiut University, Assiut, Egypt; 2 Department of Molecular Biology and Biotechnology, University of Sheffield, Sheffield, UK; 3 Institut für Radiobiologie der Bundeswehr in Verb. mit der Universitat Ulm, Neuherbergstr 11, D-80937 Munich, Germany; 4 Department of Histology, University of Navarra, Pamplona, Spain; 5 Institute of Radiation Biology, Helmholtz Zentrum München, Neuherberg, Germany

**Keywords:** 53BP1, DNA-PKcs, DSB repair, Ku70, Sertoli cells, NHEJ

## Abstract

Noncycling and terminally differentiated (TD) cells display differences in radiosensitivity and DNA damage response. Unlike other TD cells, Sertoli cells express a mixture of proliferation inducers and inhibitors in vivo and can reenter the cell cycle. Being in a G_1_-like cell cycle stage, TD Sertoli cells are expected to repair DSBs by the error-prone nonhomologous end-joining pathway (NHEJ). Recently, we have provided evidence for the involvement of Ku-dependent NHEJ in protecting testis cells from DNA damage as indicated by persistent foci of the DNA double-strand break (DSB) repair proteins phospho-H2AX, 53BP1, and phospho-ATM in TD Sertoli cells of Ku70-deficient mice. Here, we analyzed the kinetics of 53BP1 foci induction and decay up to 12 h after 0.5 Gy gamma irradiation in DNA-PKcs-deficient (*Prkdc* ^*scid*^) and wild-type Sertoli cells. In nonirradiated mice and *Prkdc* ^*scid*^ Sertoli cells displayed persistent DSBs foci in around 12 % of cells and a fivefold increase in numbers of these DSB DNA damage-related foci relative to the wild type. In irradiated mice, *Prkdc* ^*scid*^ Sertoli cells showed elevated levels of DSB-indicating foci in 82 % of cells 12 h after ionizing radiation (IR) exposure, relative to 52 % of irradiated wild-type Sertoli cells. These data indicate that Sertoli cells respond to and repair IR-induced DSBs in vivo, with repair kinetics being slow in the wild type and inefficient in *Prkdc* ^*scid*^. Applying the same dose of IR to *Prdkc* ^−/−^ and *Ku* ^−/−^ mouse embryonic fibroblast (MEF) cells revealed a delayed induction of 53BP1 DSB-indicating foci 5 min post-IR in *Prdkc* ^−/−^ cells. Inefficient DSB repair was evident 7 h post-IR in DNA-PKcs-deficient cells, but not in *Ku* ^−/−^ MEFs. Our data show that quiescent Sertoli cells repair genotoxic DSBs by DNA-PKcs-dependent NEHJ in vivo with a slower kinetics relative to somatic DNA-PKcs-deficient cells in vitro, while DNA-PKcs deficiency caused inefficient DSB repair at later time points post-IR in both conditions. These observations suggest that DNA-PKcs contributes to the fast and slow repair of DSBs by NHEJ.

**Electronic supplementary material:**

The online version of this article (doi:10.1007/s00412-016-0590-9) contains supplementary material, which is available to authorized users.

## Introduction

Of all types of DNA damage, DNA double-strand breaks (DSBs) represent the greatest threat to genome integrity. DSBs can be generated by endogenous or exogenous agents such as ionizing radiation (IR) or genotoxic chemicals. Two major pathways have been identified that repair DSBs in mammalian cells: homologous recombination (HR) and nonhomologous end joining (NHEJ) (Lieber et al. 2003). NHEJ is a rapid but error-prone repair pathway, during which the DNA-dependent protein kinase catalytic subunit (DNA-PKcs) and Ku heterodimer together form the biologically active DNA-PK holoenzyme complex that plays key roles in the repair of DSBs (Davis and Chen 2013; Gottlieb and Jackson 1993; Kakarougkas and Jeggo 2014; Meek et al. 2004). After being activated by complex formation, DNA-PKcs phosphorylates itself and other proteins such as histone H2AX and 53BP1, which are also targeted by ATM, another PI3 family kinase that responds to DSB formation (Schultz et al. 2000; Stiff et al. 2004). Microscopically, the phosphorylated histone H2AX (γ-H2AX) and the 53BP1 sensor protein form prominent visible nuclear foci at the sites of DSBs, which instigated their extensive use as DSB markers in DNA repair studies (e.g., Ahmed et al. 2012; Fernandez-Capetillo et al. 2004; Lamkowski et al. 2014; Rogakou et al. 1998). Recently, it has been shown that the time course of 53BP1 foci formation and disappearance is similar to that of γ-H2AX foci and can be used to study the DNA damage response after low and acute high doses of ionizing irradiation (IR) (Ahmed et al. 2012; Kobayashi et al. 2008; Lamkowski et al. 2014; Markova et al. 2007).

Although little information is available about the DSB repair in terminally differentiated (TD) cells in vivo, some studies addressed the DSB damage response and the radiation sensitivity in TD cells such as muscle cells, astrocytes, and other nerve cells (Narciso et al. 2007; Schneider et al. 2012). Brain neurons displayed strong 53BP1 foci formation upon irradiation, whereas adjacent TD astrocytes showed no detectable 53BP1 foci, indicating a striking difference of DNA damage response signaling between neurons and astrocytes in vivo. Therefore, different TD cell types display differences in radiosensitivity, which seem to be linked to their specific roles and physiological context (Schneider et al. 2012).

In the mammalian testis, Sertoli cells represent the supportive somatic cell lineage of the seminiferous epithelium. Sertoli cells form and expand before puberty, while postpuberty, they are unable to proliferate. Exceptions to this rule are seasonal breeders in which season-dependent variations in Sertoli cell numbers per testis occur (Johnson et al. 1991; Tarulli et al. 2006; Vergouwen et al. 1991). In the mouse, Sertoli cells proliferate until day 16 after birth (Vergouwen et al. 1991); thereafter, they permanently exit the cell cycle. Previously, both cell kinetic and radiobiological data indicated that Sertoli cells are more reminiscent of arrested proliferating cells than of classical postmitotic terminally differentiated somatic cells (Ahmed et al. 2009). Irradiation of mice has shown that Sertoli cells form 53BP1 DSB foci in vivo, which disappear with time indicating progression of DSB repair (Ahmed et al. 2007). Similar observations were made using the alkaline comet assay, showing that Sertoli cells are still able to repair IR-induced DNA breaks (Ahmed et al. 2009). Moreover, we found that Ku-dependent NHEJ is required for protecting Sertoli cells from DNA damage as indicated by the persistent foci of the DNA DSB repair-associated proteins γ-H2AX, 53BP1, and pATM in adult Sertoli cells of Ku70-deficient mice (Ahmed et al. 2013). Considering that they are in a G_1_ comparable stage of the cell cycle, adult Sertoli cells are expected to repair DSBs by error-prone NHEJ. In mice, Ku70 and Ku80 disruption leads to a hypo-fertile phenotype and deficiency in NHEJ DNA repair which is associated with early cellular senescence and compromised growth (Ahmed et al. 2013; Gu et al. 1997; Nussenzweig et al. 1997). In contrast, DNA-PKcs-deficient SCID mice are fertile, with a wild-type testicular size and no obvious deficiency in meiotic NHEJ repair (Hamer et al. 2003). Furthermore, C.B17 *Prkdc* ^*scid*^ mice express a severely hypomorphic DNA-PKcs protein (Bosma et al. 1983), which confers a twofold to threefold hypersensitivity to ionizing radiation and a deficiency in DNA DSB repair by NHEJ (Biedermann et al. 1991).

In vitro, DNA damage has been found to persist longer in *DNA-PKcs* ^−/−^ (Lobrich and Jeggo 2005), with *Ku80* ^−/−^ and *DNA-PKcs* ^−/−^ cells displaying a marked increase of irradiation-induced chromosomal aberrations (Vandersickel et al. 2010b; Virsik-Kopp et al. 2003). Moreover, previous studies have shown variable results with respect to the repair kinetics of these cells, especially shortly after IR (Iliakis et al. 2004; Reynolds et al. 2012; Vandersickel et al. 2010a). Analysis of DSB rejoining in irradiated cells using pulsed-field gel electrophoresis revealed a fast DNA repair component operating with half times of 10–30 min. This component of DSB rejoining was severely compromised in cells with mutations in DNA-PKcs, Ku, DNA ligase IV, or XRCC4, as well as after chemical inhibition of DNA-PK, suggesting the importance of DNA-PKcs and Ku for the fast repair of DSBs by the classical NHEJ pathway (reviewed in Iliakis et al. (2004) and Mladenov and Iliakis (2011)). In agreement, the repair-deficient Ku70i human cell line displayed a significantly higher number of foci up to 1 h postirradiation compared to the repair-proficient LVTHM cell line, with DSB foci numbers decreasing in both cell lines to similar background levels 24 h post IR (Vandersickel et al. 2010a). In contrast, DNA-PKcs-deficient mouse embryonic fibroblasts (MEF) showed slightly impaired repair kinetics at early time points post-IR, a more pronounced defect at intermediate times (∼4–24 h) and a dramatic defect relative to wild type, with 10 % of γ-H2AX foci persisting for 72 h or even longer (Lobrich and Jeggo 2005). On the other hand, the recruitment and loss of fluorescence intensity of DNA-PKcs-YFP over time have recently been analyzed following DSB generation by ultra-soft X-ray (USX) IR versus complex DSB induction by near-infrared photon micro-beam irradiation (Reynolds et al. 2012). It was found that Ku80 is lost within minutes from the majority of simple USX-induced DSBs, while this was not seen for DNA-PKcs, suggesting that DNA-PKcs is involved in the slow component of repair of a subset of DSBs (Reynolds et al. 2012). In all, the differences in DNA repair kinetics among DNA-PKcs and Ku-deficient cells are still not fully understood, especially in vivo shortly after DSB induction and in TD cell types.

Here, we have comparatively studied the DSB repair kinetics induced by 0.5 Gy of gamma rays by quantifying 53BP1 foci dynamics in Sertoli cells of *Prkdc* ^*scid*^ and wild-type mice as well as in MEF cell lines deficient for Ku70 and DNA-PKcs at different time points after exposure to X-irradiation. Nonirradiated Sertoli cells of *Prkdc* ^*scid*^ mice displayed elevated levels of DSBs, while IR disclosed a defective repair of IR-induced DSBs. In general, Sertoli cells displayed slower repair kinetics relative to other germ cells and MEF cells in vitro.

## Materials and methods

### Animals, irradiation, and fixation

Seven- to 8-week old males of SCID mice (C.B17, with the Icr-Prkdc SCID mutation) coding for a severely hypomorphic DNA-PKcs protein (Biedermann et al. 1991; Bosma et al. 1983), and their wild-type control were obtained from Charles River. Mice were either sham-irradiated (four mice per group) or received a whole body dose of 0.5 Gy of gamma-rays (91 MU, Elektra, Crawley, UK). Irradiated mice were sacrificed at 5 min, 1 h, 4 h, or 12 h after irradiation by CO_2_ asphyxiation. Testes were fixed in 4 % paraformaldehyde in PBS for 24 h at 4 °C. Testes were washed in 70 % EtOH prior to embedding in paraffin. Animals were kept according to approved rules of the animal welfare committee of the State of Bavaria (Az.: 55.2-1-54-2532-162-11).

### Immunohistochemistry

Testis of irradiated or sham-irradiated mice was paraffin embedded according to standard procedures, 5-μm sections were cut and mounted together on TESPA (3-aminoproyl-triethoxysilane)-coated glass slides and dried overnight at 37 °C. Sections were dewaxed in xylene and hydrated in a graded series of alcohols. For PARP1 and XRCC1 staining, sections were boiled twice for 10 min in 0.01 M sodium citrate using a microwave oven (H2500; Bio-Rad, Hercules, USA). Sections were incubated in 0.35 % H_2_O_2_ in PBS for 10 min. Blocking was done in 5 % BSA (Sigma, St. Louis, USA, A-7906) and 5 % goat serum (Vector Laboratories, S-1000, Burlingame, CA, USA) in PBS. The primary antibodies used were anti-53BP1 rabbit polyclonal (1:400; Acris Antibodies, Herford, Germany) and anti-γ-H2AX mouse monoclonal antibody (1:500, JBW301, Milipore, Germany). The slides were washed in PBS and then incubated with the secondary HRP-labeled anti-mouse/rabbit/rat (PowerVision Poly HRP; ImmunoVision Technologies, Co. Brisbane, CA, USA) for 40 min at room temperature. Bound antibodies were visualized using 0.3 g/l 3,3 diaminobenzidine (DAB, Sigma) in PBS, to which 0.03 % H_2_O_2_ was added. Sections were counterstained with Mayer’s hematoxylin. Sections were dehydrated in a series of graded alcohols and xylene and mounted with Pertex (Cellpath Ltd., Hemel Hempstead, UK).

### Cell lines culture and irradiation

Wild-type, *DNA-PKcs* ^−/−^, and *Ku* ^−/−^ mouse embryonic fibroblast cell lines (Araki et al. 1999) were kindly provided by Dr. D.J. Chen (Southwestern Medical Center, Dallas, USA). Cells were cultured as monolayers in growth medium consisting DMEM/F12 and 5 % bovine serum, in a humidified 5 % CO_2_ incubator at 37 °C. To induce cell cycle synchronization, the growing cell lines were washed twice in phosphate buffer saline (PBS, pH 7.4), trypsinized and re-suspended in medium (DMEM F12) without serum at a concentration of 0.5 × 10^6^ cells/ml. Cells from wild-type, *DNA-PKcs* ^−/−^, and *Ku* ^−/−^ were cultured in three well plates (each plate containing one cell line) and serum-starved for 18 h before irradiation. Irradiation was done with 240 kV X-rays at 13 mA, filtered with 3 mm beryllium at 1 Gy/min using a YXLON Maxishot (Hamburg, Germany) device. After irradiation with 0.5 Gy, cell cultures were further incubated for 5 min, 30 min, 1 h, 3 h, and 7 h. At the respective time points, sham-irradiated and X-irradiated cell lines were washed in PBS and fixed in 80 % ice-cold methanol for 1 min. Cells were covered with 70 % ethanol and kept at −20 °C until immunofluorescent staining.

### Flow cytometry

For flow cytometry, cells from the control and the irradiated samples were washed in PBS and trypsinized with 0.5 % trypsin in PBS. Then, the pellets were collected by centrifugation (5000 rpm/min), resuspended in 70 % cold ethanol, and kept at −20 °C. The cell cycle analysis by flow cytometry was done as described previously (Muradyan et al. 2011) using a FACSCalibur flow cytometry (Becton Dickinson). The cellular DNA content was analyzed using propidium iodine (PI)-stained nuclei. The distribution of cells in G_1_, S, and G2/M phase was estimated using CellQest software (Becton Dickinson). The DNA index of the three MEF cell lines was determined using the same FACS setup and DAPI staining.

### Immunofluorescence, fluorescence in situ hybridization, and image analysis

Slides of control and irradiated cell lines were permeabilized in ice-cold PBS containing 0.2 % Triton X-100 for 10 min. Cells were washed with PBS and blocking was done in PBS/0.1 % Tween 20/0.2 % BSA/0.1 % fish gelatin (PBTG) buffer. The slides were incubated with the primary antibodies for 1 h at 37 °C in PBTG buffer, followed by 3 × 5-min washes in PBS and incubation with the secondary antibodies for 45 min. The primary antibodies used were mouse monoclonal anti-proliferating cell nuclear antigen (PCNA; Merck) (1:200 in blocking buffer), rabbit polyclonal anti-53BP1 (1:400; Acris Antibodies), and mouse monoclonal antibody anti-γ-H2AX (1:500, JBW301, Millipore). After washes in PBTG and incubation with the secondary antibodies, sections were again washed three times 5 min in PBS at 37 °C. The secondary antibodies were goat anti-mouse Alexa 488 and 516 (1:800, Dianova) and Donkey anti-rabbit-Cy3 (1:800, Dianova). Slides were supplied with 18ul Vectashield Mounting Medium (Vector labs) containing DAPI as DNA/nuclear counterstain and covered with a 24 × 60-mm cover slip. Preparations were analyzed using a motorized Zeiss Axioplan 2 fluorescence microscope equipped with the ISIS fluorescence imaging system (MetaSystems, Altlussheim). Digital images of several optical planes of the sections were recorded and combined to a maximum projection images that were manually analyzed for the presence of foci/nucleus or for the presence of cells.

### Telomere FISH

Telomere fluorescence in situ hybridization (FISH) using a (CCCTAA)_7_ (TTAGGG)_7_ telomere repeat probe (DAKO Cytomation, Denmark) was carried out as described in detail elsewhere (Liebe et al. 2006; Scherthan 2009).

### TUNEL assay

TUNEL analysis was performed to detect apoptotic nuclei in 5-mm paraffin-embedded sections (Ahmed et al. 2013) according to the manufacturer’s protocol (In Situ Cell Death Detection Kit, POD; Roche Diagnostics GmbH, Mannheim, Germany).

### Statistical Analysis

The results were analyzed using *t* test and the data were expressed as mean ± standard deviation (SD) using GraphPad software (graphpad.com). Fifty to 100 cells per time point and experiment were analyzed, with the experiments being repeated three times.

## Results

### DNA-PKcs-deficient SCID mice Sertoli cells display persistent DSBs foci

**Fig. 1:**
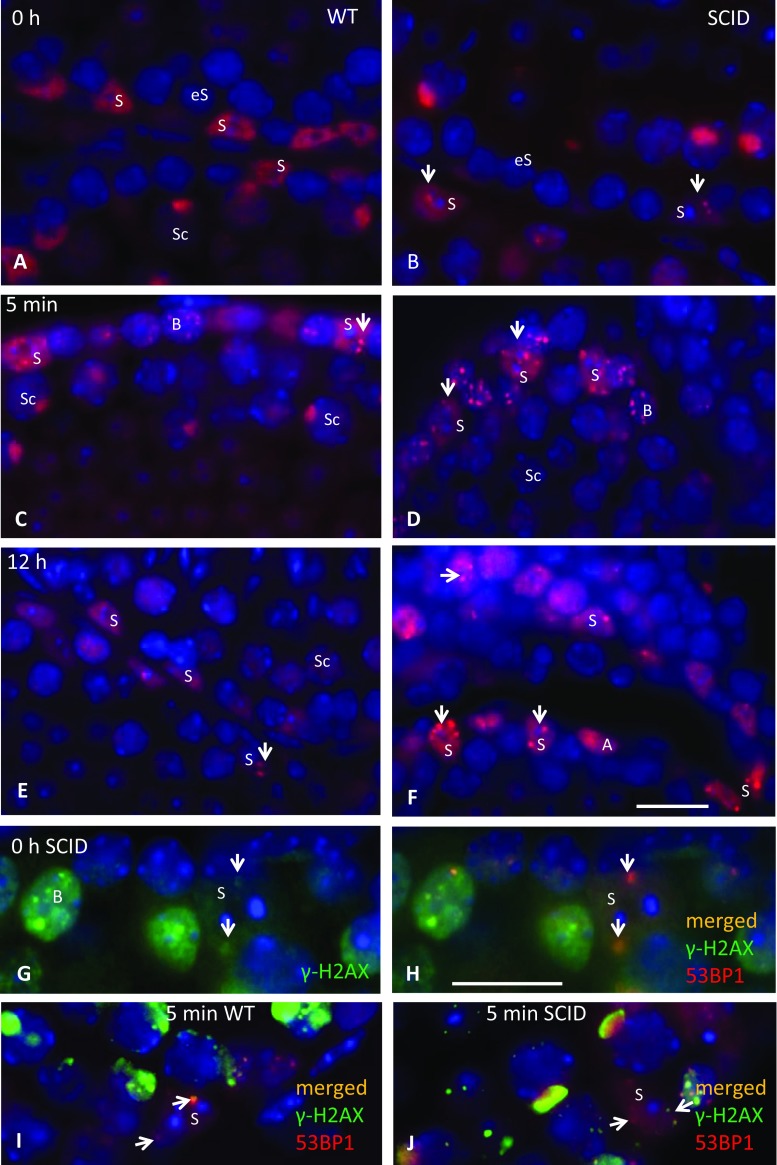
DSB damage persistence before IR and repair kinetics after IR in Sertoli cells. **a** Testicular section of a wild-type mouse showing no 53BP1 DSB-indicating foci in Sertoli cells. Sertoli cells are characterized by irregular-shaped nucleus with two *blue dots* next to the *dark spot* (the nucleolus) representing the chromocenters that are specific for this cell type. **b** 53BP1 foci (*red*) in *Prdkc* ^*scid*^ Sertoli cells (*arrows*). **c–f** Representative images for IR-induced 53BP1 foci in wild-type and SCID Sertoli cells. **g**, **h** γ-H2AX foci in nonirradiated *Prdkc* ^*scid*^ Sertoli cells (*arrows* in G indicate *green* γ-H2AX foci and *arrows* in H show co-localized foci). **i** Irradiated wild-type testis (*arrows* show co-localized foci). **j** *Prdkc* ^*scid*^ Sertoli cells displaying γH2AX foci co-localized with 53BP1 (*arrows*). *S* Sertoli, *Sc* spermatocyte, *B* type B spermatogonia, *A* type A spermatogonia, *eS* early spermatocytes. *Scale bars* represent 10 μm

**Fig. 2:**
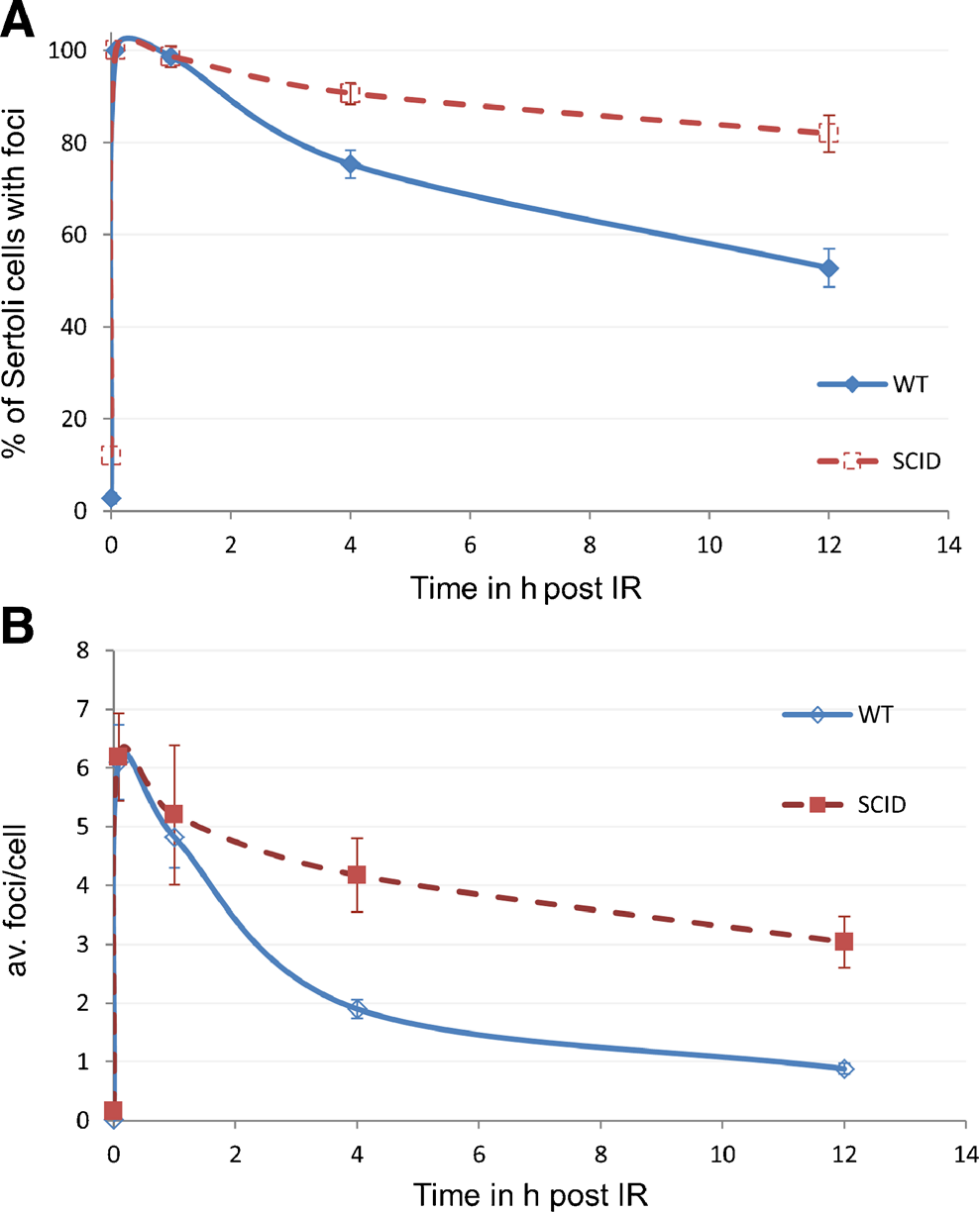
DNA repair kinetics after IR in Sertoli cells of wild-type and *Prdkc* ^*scid*^ mice. **a** Percentages of Sertoli cells with foci at different time points after 0.5 Gy gamma-IR. **a** Average number of 53BP1 foci per Sertoli cell before and at different time points after IR. Fifty cells per mouse were analyzed per time point (three mice each)

Recently, we observed that adult Sertoli cells of Ku70-deficient mice displayed γ-H2AX, 53BP1, and p-ATM DSB foci indicating that NHEJ may be protecting Sertoli cells from DNA damage (Ahmed et al. 2013). To further investigate the involvement of NHEJ in protection of adult Sertoli cells from DNA damage, here we checked the presence of 53BP1 DSB-indicating foci in nonirradiated and irradiated *Prkdc* ^*scid*^ mouse Sertoli cells. In nonirradiated *Prkdc* ^*scid*^ mice, about 12 % of Sertoli cells showed one to three large 53BP1 foci per cell (Fig. 1a, b), representing a significant increase of the average foci per cell (fpc) number relative to wild-type Sertoli cells that displayed only a few spontaneous foci (Figs. 1a, b and 2b). 53BP1 foci also co-localized with γ-H2AX foci (Fig. 1g, h) likely indicating true DSBs. By comparing the persistent foci in Sertoli cells from nonirradiated *Prkdc* ^*scid*^ and Ku70-deficient mice, the latter showed about twofold increase in both the average fpc and the percentages of cells with foci (Table 1, Fig. 2). These data indicate that the persistent foci phenotype in *Prkdc* ^*scid*^ Sertoli cells is less severe compared to that of Ku70-deficient mice. This stresses that both DNA-PKcs and Ku70 (the main components of the classical NHEJ) are required for protecting Sertoli cells from DNA damage.

**Table 1:** Frequency of persistent DSBs foci in Sertoli cells of nonirradiated *Prdkc* ^*scid*^ and Ku70-deficient mice

53BP1 foci in un-irradiated Sertoli cells	WT	SCID	*Ku70* ^−/−^
Number of foci/cell	0.03 ± 0.03	0.17 ± 0.03 ^a^	0.33 ± 0.15 ^a^
% of cells with foci	2.6 ± 1.2 %	12 ± 2 % ^a^	25.1 ± 10.3 % ^a^

Foci data of *Ku70* ^−/−^ mice were taken from (Ahmed et al. 2013)

### Inefficient repair of IR-induced DSBs in SCID Sertoli cells

To further investigate the DSB repair efficiency in vivo in Sertoli cells, we analyzed the kinetics of 53BP1 foci induction and removal in 0.5 Gy of gamma irradiation-exposed wild-type and *Prkdc* ^*scid*^ Sertoli cells (Fig. 1c–f). The observed IR-induced foci also co-localized with γ-H2AX foci in wild-type and *Prkdc* ^*scid*^ Sertoli cells (Fig. 1i, j) indicating the sites of DSB formation. To test for cell death effects after IR exposure, we stained testes sections for apoptotic cells by the TUNEL assay—no elevated apoptosis rates before and after IR were observed in wild-type and *Prkdc* ^*scid*^ testes (Suppl. Fig. 2). The Sertoli cells from wild-type and *Prkdc* ^*scid*^ mice displayed on average six foci (range 1–13 fpc) 5 min after IR (Figs. 1 and 2). There was no significant difference between the two groups at this early time point (Fig. 2b). Wild-type Sertoli cells managed to repair around two-thirds of IR-induced foci within 4 h post-IR and still displayed 1–3 foci in 73 and 52 % of cells, 4 and 12 h post-IR, respectively. Then, Even after 12 h post 0.5 Gy (which is a relatively low dose), residual IRIF were detected in the wild type. *Prkdc* ^*scid*^ Sertoli cells needed 12 h to repair around 50 % of the induced DSBs with more than 90 and 80 % of cells at 4 and 12 h post-IR, respectively, still showing persistent 53BP1 foci (Fig. 2). Together, these data indicate that Sertoli cells respond to and repair IR-induced DSBs in vivo, with the repair kinetics being slow in the wild type and inefficient in SCID (NHEJ compromised).

### DNA damage foci and telomeres in SCID Sertoli nuclei

**Fig. 3:**
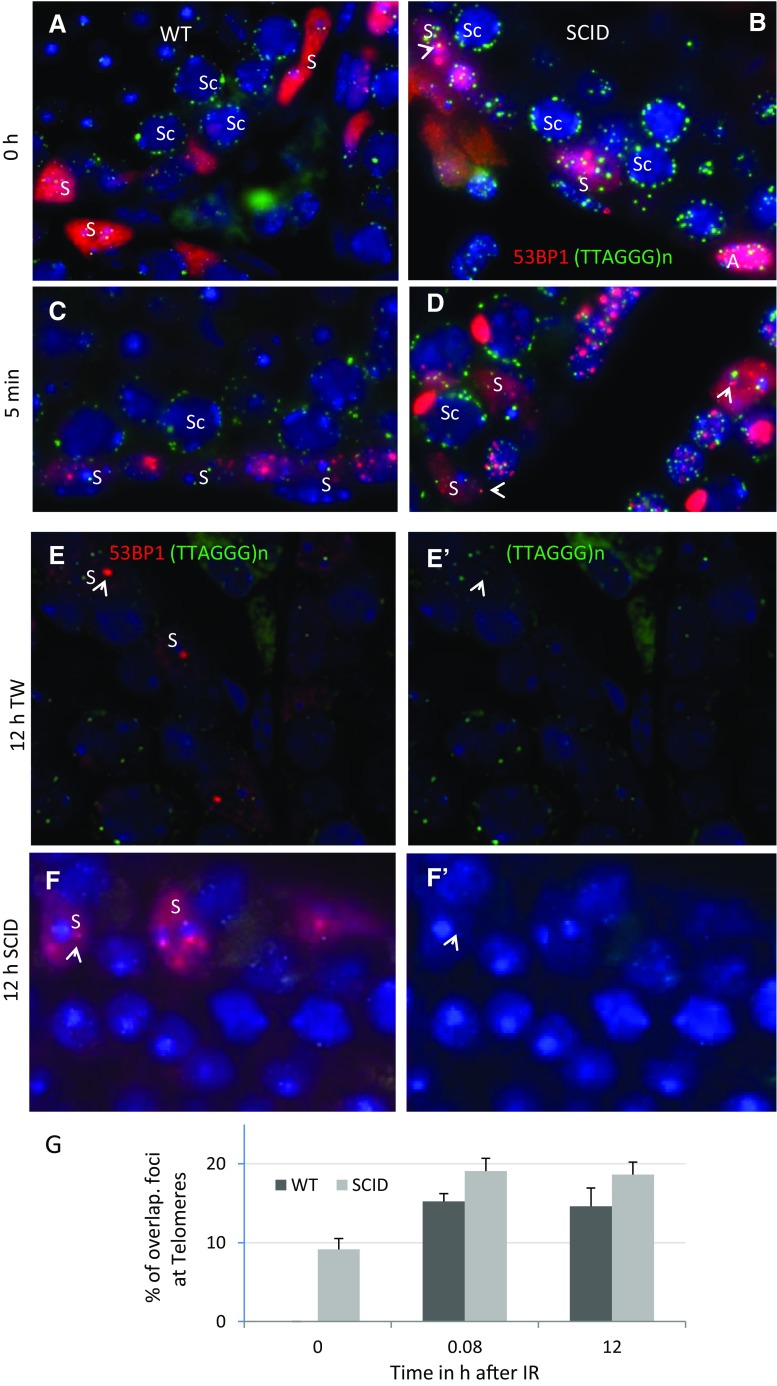
Telomeres and DSB damage foci at Sertoli cells before and after IR. **a**–**f** Representative images form nonirradiated **a**, **b** and irradiated **d**–**f** wild-type and *Prdkc* ^*scid*^ testes showing the co-localization of 53BP1 foci at telomeres of TD Sertoli cells (*arrow heads*). **g** Percentages of 53BP1 foci that overlap (partially or co-localize) with telomere signals before and after IR. *S* Sertoli, *Sc* spermatocyte, *B* type B spermatogonia, *A* type A spermatogonia, *eS* early spermatocytes. *Scale bars* at 10 μm

The DNA-PKcs enzyme is required for efficient DSB repair and also has been implied in telomeric end protection (Bailey et al. 2004). Thus, we checked whether persistent 53BP1 DNA damage foci in *Prkdc* ^*scid*^ Sertoli nuclei co-localize with TTAGGG_n_ telomeres. To this end, testicular sections were co-stained for 53BP1 by IF and telomeres by TTAGGG-FISH. 53BP1 DSB damage foci were observed to partially overlap with the telomere FISH signals in 8.8 % (±1.6 SD) of *Prkdc* ^*scid*^ Sertoli nuclei (Fig. 3, arrows head). In irradiated Sertoli cells, the percentages of partially overlapped 53BP1 foci with telomere FISH signals were 14.9 % (±1.3) 5 min post-IR and 15 % (±1.1) 12 h post-IR in wild-type Sertoli cells. In *Prkdc* ^*scid*^ Sertoli cells, these percentages were 18.7 % (±2.4) 5 min post-IR and 17.9 % (±1.4) 12 h post-IR, indicating a slight but insignificant (*p* = 0.45) increase among irradiated *Prkdc* ^*scid*^ and wild-type Sertoli cells (Fig. 3g). In all, there was no significant difference between *Prkdc* ^*scid*^ and wild-type mice, indicating that persistent DSB damage foci in Sertoli cells are not due to deprotection of telomeres.

### Cell cycle and DNA repair kinetics analysis of NHEJ-deficient MEFs

**Fig. 4:**
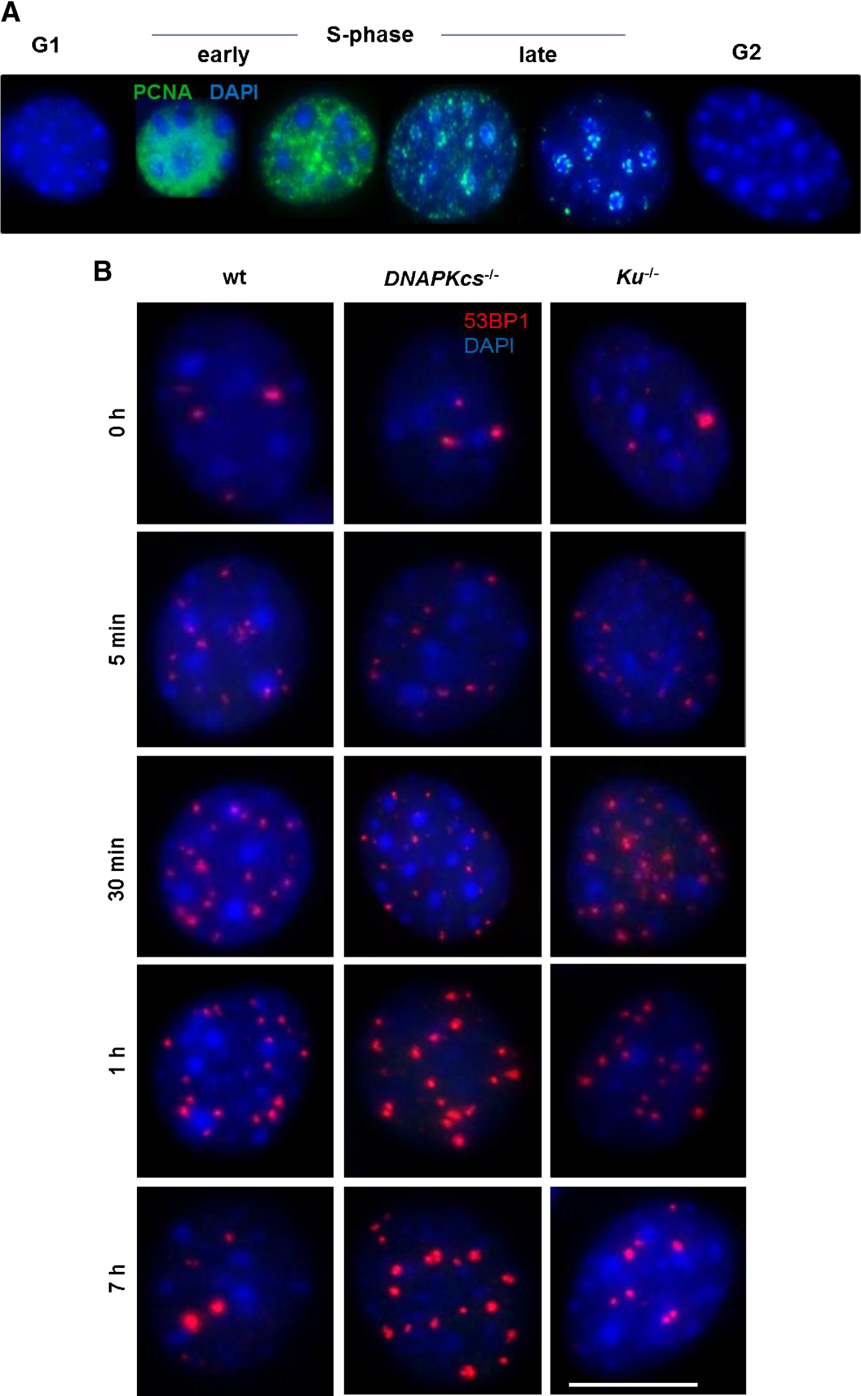
Immunofluorescent analysis of different cell cycle phases in 53BP1-stained wild-type, *DNA-PKcs* ^−/−^ and *Ku* ^−/−^ MEF cell lines. Cells were synchronized by serum starvation for 18 h. **a** Substages of S-phase cells shown according to PCNA (*green*) staining patterns; G_1_ and G_2_/M phases are negative for PCNA. **b** Representative images showing the presence, induction, and disappearance of 53BP1 DSB-indicating foci after IR in wild-type, *DNA-PKcs* ^−/−^, and *Ku* ^−/−^ MEF cells 0 h, 5 min, 30 min, 1 h, and 7 h post-IR. Note the increase in foci after 7 h of IR in *DNA-PKcs* ^−/−^ cells

We next comparatively studied IR-induced foci formation and their decay in wild-type, *DNA-PKcs* ^−/−^, and *Ku* ^−/−^ MEF cell lines. We applied serum starvation to enrich for G_1_ phase cells. Eighteen hours of serum starvation was necessary to reduce the high level of background foci levels in cultured wild-type, *DNA-PKcs* ^−/−^, and *Ku* ^−/−^ MEF cell lines (Fig. 4). After IF-staining with the PCNA S-phase marker, microscopic patterns in control and irradiated cells were used to address the different phases of the cell cycle (Fig. 4a). S-phase and G_2_-phase cells were excluded from the foci analysis by excluding PCNA-positive cells and cells with large DAPI-bright heterochromatin clusters (these are enlarged after DNA replication [G_2_ phase]). Flow cytometry analysis of PI-stained control and irradiated cell lines showed that more than 56 % of cells were in G_1_ phase, and less than 20 % of cells were in S-phase in all tested cell lines and time points (Suppl Fig. 1).

**Fig. 5:**
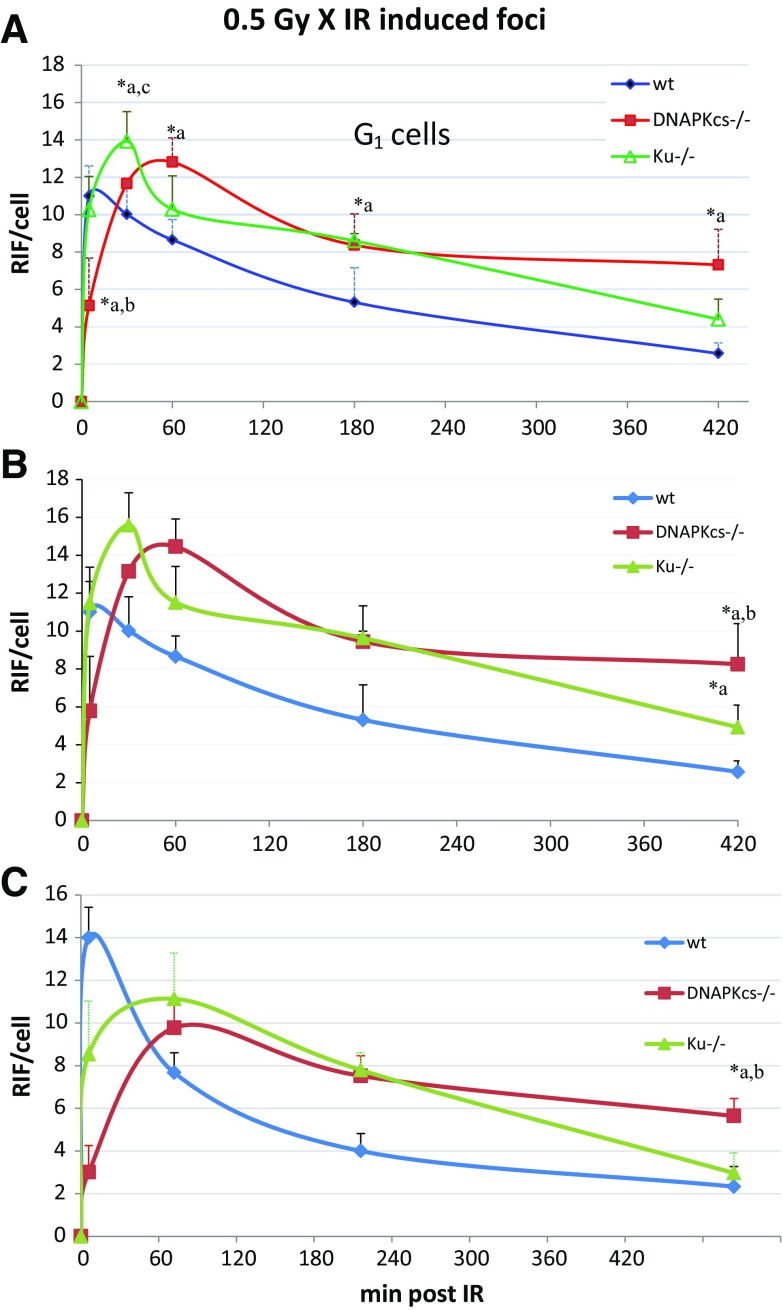
Radiation-induced 53BP1 foci (RIF) in wild-type and mutant MEF cell lines. **a** The kinetics of induction and loss of 53BP1 foci in sham-irradiated and X-irradiated wild-type, *DNA-PKcs* ^−/−^, and *Ku* ^−/−^ MEFs (in G_1_-phase) 5 min, 30 min, 1 h, 3 h, and 7 h post-IR. **b** Repair kinetics after applying a correction factor based on the FACS analysis and the DI of cell lines. **c** Repair kinetics in S-phase cells and background foci (at 0 h) were subtracted and foci values were normalized according to the DI. Compared to the wild type (*a*), compared to the *Ku* ^−/−^ (*b*), and compared to *DNA-PKcs* ^−/−^ (*c*). *p < 0.05

By applying the same 0.5Gy dose of X-irradiation to MEF cells, we quantified the kinetics of induction and loss of 53BP1 foci in sham-irradiated and X-irradiated wild-type, *DNA-PKcs* ^−/−^, and *Ku* ^−/−^ MEFs 5 min, 30 min, 1 h, 3 h, and 7 h post-IR. Representative images for the control and the irradiated cells stained for 53BP1 and PCNA are shown in Fig. 4. After subtracting the background foci, an average of 11 radiation-induced foci per cell were seen in wild-type MEFs 5 min after in vitro IR, which is almost twofold more than foci seen in vivo in adult Sertoli cells 5 min after IR and indicates the difference of in vivo and in vitro systems. Our repair kinetics data show significant DSB surrogate foci induction in all cell lines 5 min post 0.5-Gy X-irradiation (Fig. 5), with the number of 53BP1 foci being significantly lower in DNA-PKcs-deficient cells compared to wild-type (*p* = 0.028) and Ku-deficient cells (*p* = 0.046), indicating a delay in the induction of 53BP1 foci in *DNA-PKcs* ^−/−^ cells during the early response to IR-induced DSBs. Foci numbers 30 min post-IR showed no significant differences between the DNA-PKcs-deficient cells and the wild type, while an elevated number of foci was obtained in this genotype 1, 3, and 7 h post-IR, with the increase being significant relative to wild type at time points 1 and 7 h after IR (*p* = 0.013 and 0.014, respectively). These data indicate a delay in the induction of 53BP1 foci in *DNA-PKcs* ^−/−^ cells 5 min post-IR, which is in contrast to *Prkdc* ^*scid*^ Sertoli cells that showed no clear difference to the wild type within 5 min of IR. Results also show inefficient repair at more advanced time points post-IR in DNA-PKcs-deficient cells, indicating that DNA-PKcs contributes to the slow component of DSB repair.

*Ku* ^−/−^ cells on the other hand displayed significantly increased fpc numbers 30 min after in vitro IR, relative to the wild-type (*p* = 0,047), while the difference to *DNA-PKcs* ^−/−^ cells being insignificant. Wild-type cells reached the maximum fpc values after 5 min, while *Ku* ^*−/−*^ *and DNA-PKcs* ^−/−^ cells displayed max foci values 30 and 60 min after irradiation, respectively (Fig. 5), indicating that NHEJ-deficient cells require more time for full focus formation with *DNA-PKcs*-deficient cells being most severely affected. In *Ku* ^−/−^ cells, the average 53BP1 fpc numbers were only slightly higher relative to the wild-type 3 h post irradiation (*p* = 0.039) but had reached nearly wild-type level 7 h post-IR (*p* = 0.06). These data indicate that the NHEJ cells display a DSB repair capacity similar to the wild type within a few hours of exposure to a low dose of 240 kV X-irradiation. The data also are in agreement with the findings that Ku is predominantly required for the fast component of NHEJ DSB repair and that DNA-PKcs contributes to both, DSB foci formation and repair outcome (Vandersickel et al. 2010a).

Since MEF cell lines tend to become aneuploidy upon prolonged culture, we also controlled for ploidy changes and determined the DNA index (DI) for all lines by FACS analysis with human lymphocytes as diploid control. It was found that all lines are aneuploid, with a DI of 1.53 for wild type, 1.67 for DNA-PKcs−/−, and a DI of 0.93 for 25 % of cells and 1.71 for 75 % of cells in the *Ku* ^*−/−*^ line, respectively. We therefore normalized the foci values to the DI of 1.67 by applying a correction factor based on the assumption that 1 Gy of X-irradiation will create approx. 40 DSBs (Ward 1991) and that larger genomes will receive proportionally more DSBs. However, using normalization or not revealed similar results in the lines investigated (Fig. 5b).

Moreover, we have also quantified the DSB repair kinetics in irradiated MEFs in S-phase stage after subtracting the background foci in sham-irradiated cells (Fig. 5c). Interestingly, similar repair kinetics to the non S-phase cells were seen especially at more advanced time points after IR.

## Discussion

Here, we investigated the presence of 53BP1 foci as DSB surrogate marker in untreated *Prdkc* ^*scid*^ Sertoli cells in order to check the involvement of DNA-PKcs in the protection of Sertoli cells from DNA damage, as recently noted for the Ku70-deficient testis (Ahmed et al. 2013). We also comparatively studied the kinetics of foci disappearance (repair) after IR of *Prdkc* ^*scid*^ mice and their wild-type control in vivo and in MEFs in vitro.

Adult *Prdkc* ^*scid*^ Sertoli cells showed a >fivefold significant increase in the average number of DSB-indicating foci relative to wild type, suggesting that the DNA-PK-dependent NHEJ repair pathway is operating in adult Sertoli cells. Comparing the average fpc and the percentage of Sertoli cells carrying foci in *Prdkc* ^*scid*^ and *Ku70* ^−/−^ testes sections indicates that the later phenotype is more severe, which is probably due to a DNA-PKcs residual activity in the *Prdkc* ^*scid*^ cells used (Beamish et al. 2000; Woo et al. 1998). Furthermore, TD Sertoli cells may also use the alternative NHEJ pathway to rejoin DSBs, by action of the synaptic activity of PARP1 and the ligation activity of the XRCC1-DNA ligase III complex (Audebert et al. 2004). However, PARP1-inhibited Sertoli cells failed to display persistent foci for 53BP1 or γ-H2AX DSB markers (Ahmed et al. 2010). Together, these data strongly suggest that cNHEJ protects adult TD Sertoli cells from accumulation of dsDNA damage.

We also found that TD Sertoli cells respond to IR-induced DSBs in vivo by forming DSB-indicating foci, which were only 50 % of the number of foci induced in vitro in G_1_ MEFs after exposure to the same dose. This may likely relate to cell type and in vivo/vitro differences, since Sertoli cells have a completely different heterochromatin distribution and nuclear organization compared to fibroblasts (Bridger et al. 2000; Mayer et al. 2005; Scherthan et al. 2000). Our data revealed ∼6 foci/Sertoli cell 5 min post-IR implying fast repair; this may indicate that around 12 DSBs/Gy were formed in Sertoli cells in vivo directly after IR. For human fibroblasts in the G_1_ phase of the cell cycle PFGE DSB estimation revealed about 25 DSBs/Gy (Lobrich et al. 2010), which contrasts with a detection level of around 1 in 2 DSBs per Sertoli cell in in vivo analysis. Moreover, our in vitro data about the induced DSBs/Gy at earliest time point after IR are consistent with the estimated numbers by physical methods (Lobrich et al. 2010).

Sertoli cells in the seminiferous tubules of *Prdkc* ^*scid*^ and WT testes were found to form 53BP1 foci 5 min post 0.5-Gy gamma-irradiation, while >66 % of DSBs were repaired within 12 h post-IR. However, around 50 % of cells failed to repair all DSBs after 12 h of IR, which is surprising for this relativity low dose. The persistence of foci was not correlated with elevated apoptosis (Suppl. Fig. 2), indicating that persistent unrepairable damage did not induce apoptosis in damage-carrying Sertoli cells. This is in accordance with the accumulation of regions of unrepairable complex DNA damage in tissues in vivo, especially such with cells with long life spans that are resistant to apoptosis, like cells in the ageing brain, pancreas, skin, and kidney (Noda et al. 2012; Ahmed et al. 2012).

The repair of IR-induced DSBs in *Prdkc* ^*scid*^ Sertoli cells in vivo was slower and less efficient, since 80 % of cells still showed foci after 12 h post-IR. Similar observations were made in TD astrocytes that showed a significantly impaired DSB repair when both DNA-PKcs and ATM were inhibited (Schneider et al. 2012). Furthermore, the terminal differentiation of 3T3-T cells reduced the repair of IR-induced DNA DSBs (Bill et al. 1992). Moreover, our previous unpublished observations showed no significant variation in persistent 53BP1 foci (after 8 h of IR) in Sertoli cells of wild-type and PARP1-inhibited mice that expressed PARP1 and XRCC1 in Sertoli cells before after IR (Ahmed et al. 2010). Together, these data indicate that DNA-PKcs-dependent NHEJ is required for the slow and the efficient repair of DSB in adult TD Sertoli cells.

53BP1 fpc numbers in the DNA-PKcs-deficient cell line were significantly lower compared to the wild-type and *Ku70* ^−/−^ cells 5 min after IR, indicating a requirement for the DNA-PKcs kinase for full DNA damage signaling early after DSB formation. 53BP1 and γ-H2AX foci formation was detected as early as 1–5 min after IR (Bekker-Jensen et al. 2005; Rogakou et al. 1999; Rothkamm et al. 2003). In *Ku* ^−/−^ cell line cells, we noted a significant increase of 53BP1 foci relative to wild-type and the *DNA-PKcs* ^−/−^ cell line 30 min post-IR, indicating the expected contribution of Ku70 to the fast DSB repair component after irradiation (Iliakis et al. 2004; Vandersickel et al. 2010a). Still, Ku-deficient cells required slightly more time to reach the full foci values, underlining Ku’s role in DNA-PKcs recruitment to DSBs (Uematsu et al. 2007).

The delay in the induction of 53BP1 foci 5 min post-IR in MEFs was in contrast to in vivo *Prdkc* ^*scid*^ Sertoli cells that showed no clear difference to the wild type within 5 min of IR. DNA-PKcs knockout mice have been found to display phenotypes similar to the SCID mouse (Beamish et al. 2000; Gao et al. 1998; Taccioli et al. 1998). Here, our DSBs repair kinetics data revealed a delay in the early response to IR-induced 53BP1 foci in MEFs cell lines but not in *Prdkc* ^*scid*^ Sertoli cells, which is probably related to a residual kinase activity in the SCID mice used (Woo et al. 1998; Beamish et al. 2000). It has been observed that in the absence of DNA-PKcs activity, ATM is downregulated (Peng et al. 2005) leading in turn to a delayed foci response. Moreover, PARP inhibition has been reported to block DSB repair in Ku-deficient but not in DNA-Pkcs-deficient cells (Veuger et al. 2004; Wang et al. 2006). While the observed efficient DSB repair 7 h post-IR in our *Ku* ^−/−^ but not in DNA-PKcs-deficient cells may in part reflect cell line-specific fluctuations, it may also relate to the ability of Alt-NHEJ to proceed in the absence of Ku but not in the absence of DNA-PKcs (Wang et al. 2006).

## Conclusions

Our data highlight the requirement of DNA-PKcs for protection of TD Sertoli cells from dsDNA damage that may result from oxidative stress or from exogenous noxes like IR exposure. Moreover, our in vivo and in vitro data support the view that DNA-PKcs is involved in both the fast and the slow repair component of cNHEJ.

## Electronic supplementary material

Below is the link to the electronic supplementary material.

ESM 1 (PDF 1159 kb)
