# Supplementary material for: DNA repair kinetics in SCID mice Sertoli cells and DNA-PKcs-deficient mouse embryonic fibroblasts
Source: Chromosoma. 2016 May 2;126(2):287–98. doi: 10.1007/s00412-016-0590-9 (PMC5371645; doi:10.1007/s00412-016-0590-9)
Supplement: Supplementary file 1 — (PDF 1159 kb) [file 412_2016_590_MOESM1_ESM.pdf]

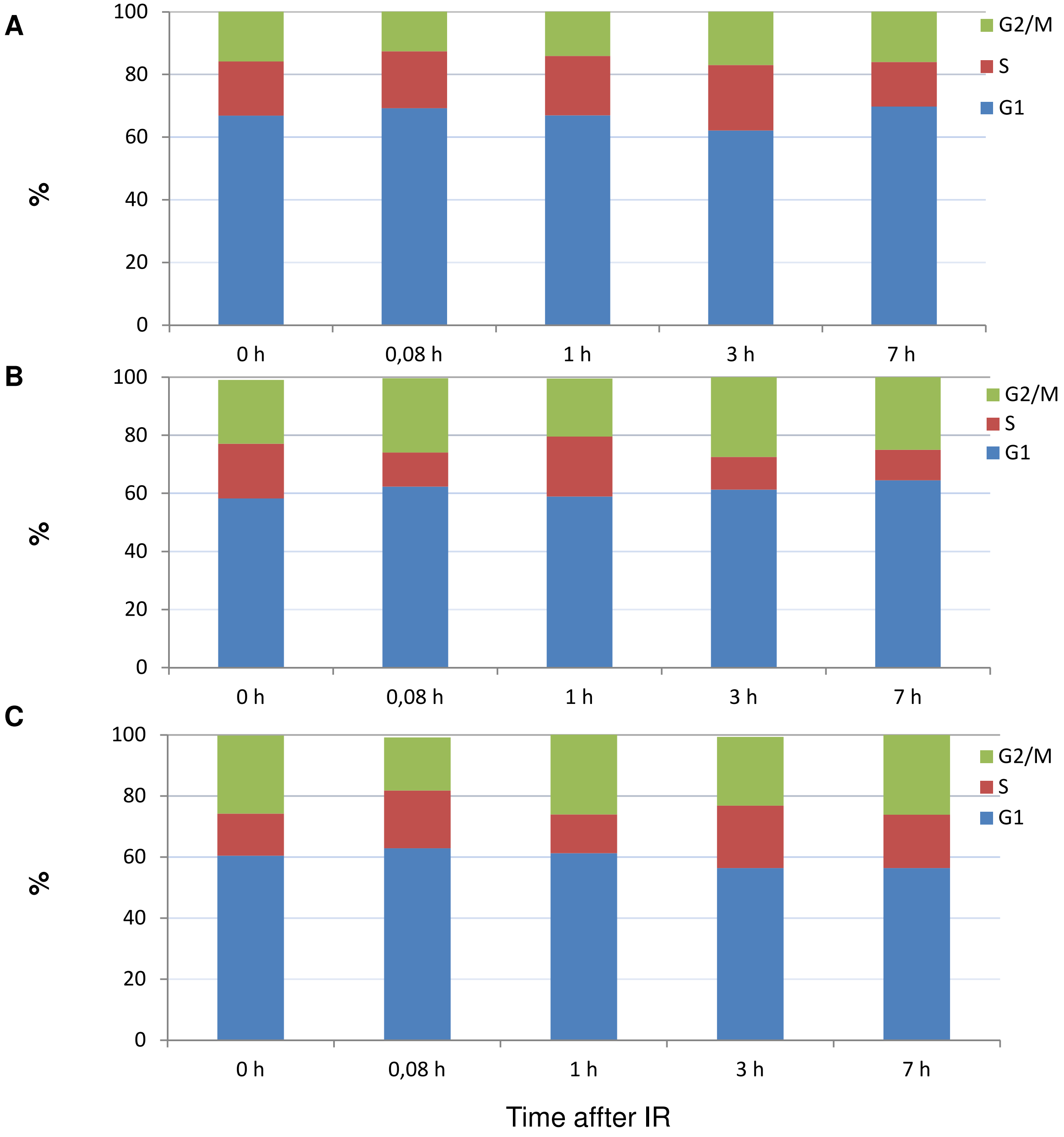

Suppl. Fig. 1. Flow cytometry analysis of cell cycle stages of PI-stained control and irradiated cell lines 5 min, 1 hr, 3 hr and 7 hr post IR, (A) *WT* , *(B) DNAPKcs*$^{-/-}$ (C) *Ku*$^{-/-}$. S-phase cells were less than 20% in all cell lines.

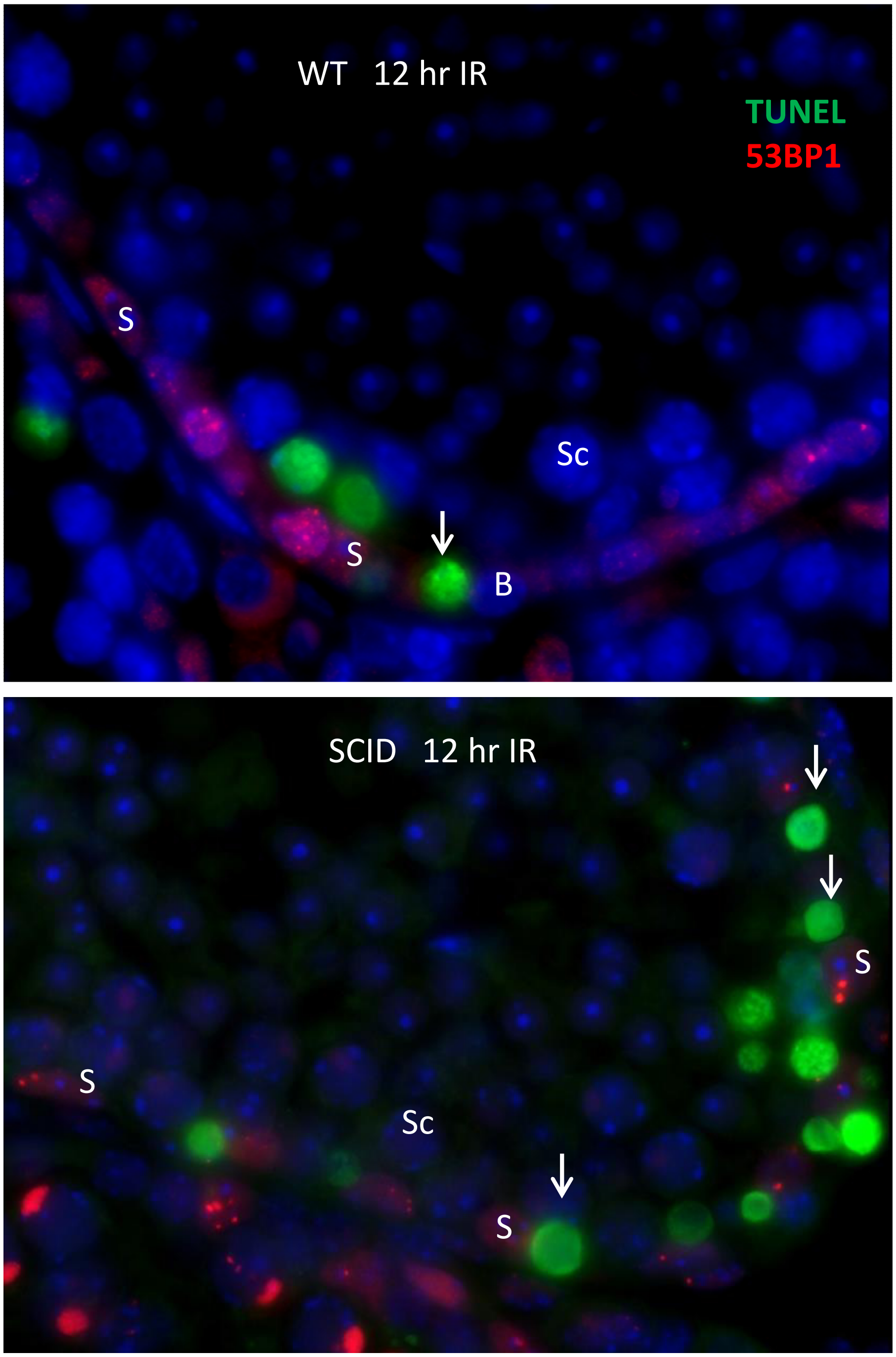

Suppl Fig. 2. Testis tubule sections in WT and SCID mice stained for apoptosis (TUNEL) and 53BP1 revealing that, in contrast Spermatogonia, Sertoli cells are resistant to IR-induced apoptosis. S, Sertoli; Sc, spermatocyte; B, type B spermatogonia.

Fig. 4.

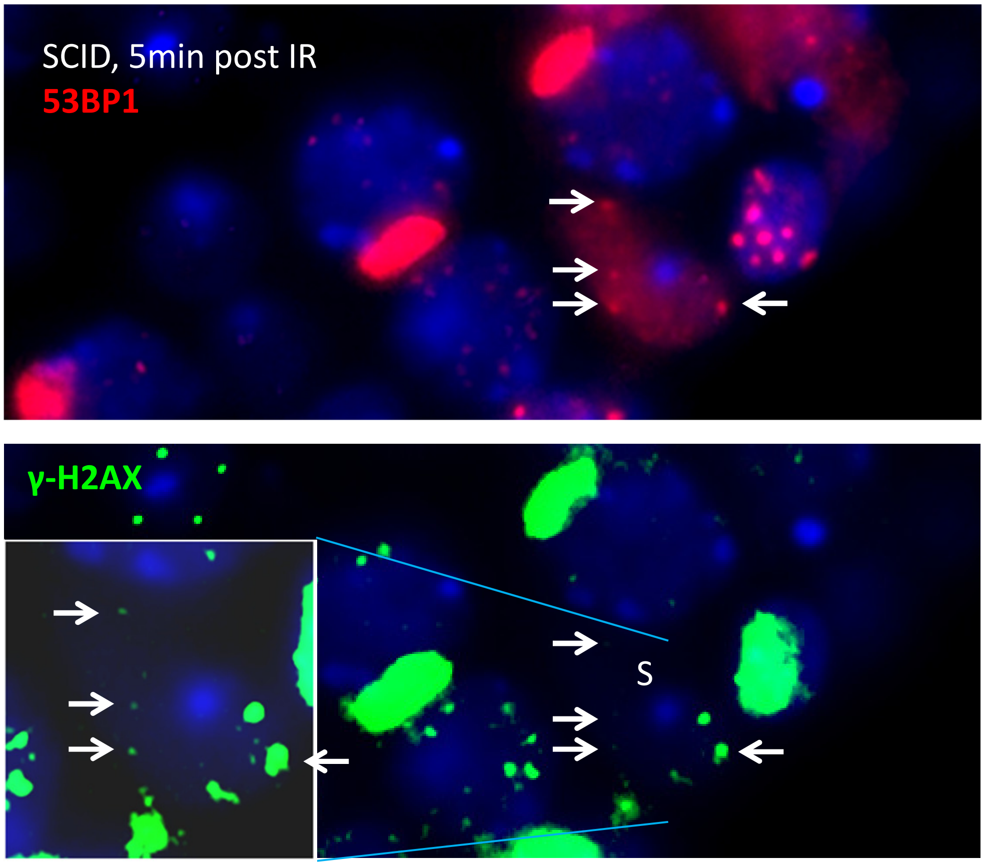

Suppl Fig. 3. Split color channels of Fig. 1J showing the co-localization of 53BP1 foci and γ-H2AX foci in Sertoli cells (S). Inset: enhanced detail of the Sertoli cell to the right showing the small γ-H2AX foci at 53PB1 foci indicated above (arrows). The bright staining of the XY body and two γ-H2AX foci (arrows) resulted from increased camera exposure time to be able to reveal the weaker γ-H2AX foci in Sertoli cells.
